# A germline mutation of *CDKN2A* and a novel *RPLP1-C19MC* fusion detected in a rare melanotic neuroectodermal tumor of infancy: a case report

**DOI:** 10.1186/s12885-016-2669-3

**Published:** 2016-08-12

**Authors:** David J. Barnes, Edward Hookway, Nick Athanasou, Takeshi Kashima, Udo Oppermann, Simon Hughes, Daniel Swan, Dietrich Lueerssen, John Anson, A. Bassim Hassan

**Affiliations:** 1 Nuffield Department of Orthopaedics, Rheumatology and Musculoskeletal Sciences, University of Oxford, Nuffield Orthopaedic Centre, Windmill Road, Headington, Oxford OX3 7HE UK; 2 Oxford Gene Technology Ltd, Begbroke Science Park, Begbroke Hill, Woodstock Road, Begbroke, Oxfordshire OX5 1PF UK; 3 Tumour Growth Group, Oxford Molecular Pathology Institute, Sir William Dunn School of Pathology, University of Oxford, South Parks Road, Oxford, OX1 3RE UK

**Keywords:** Melanotic Neuroectodermal Tumor of Infancy, Germline CDKN2A mutation, RPLP1-C19MC fusion gene, RNA-Seq, Sensitivity to lysine demethylase inhibitors

## Abstract

**Background:**

Melanotic neuroectodermal tumor of infancy (MNTI) is exceptionally rare and occurs predominantly in the head and neck (92.8 % cases). The patient reported here is only the eighth case of MNTI presenting in an extremity, and the first reported in the fibula.

**Case presentation:**

A 2-month-old female presented with a mass arising in the fibula. Exhaustive genomic, transcriptomic, epigenetic and pathological characterization was performed on the excised primary tumor and a derived cell line. Whole-exome analysis of genomic DNA from both the tumor and blood indicated no somatic, non-synonymous coding mutations within the tumor, but a heterozygous, unique germline, loss of function mutation in *CDKN2A* (p16^INK4A^, D74A). SNP-array CGH on DNA samples revealed the tumor to be euploid, with no detectable gene copy number variants. Multiple chromosomal translocations were identified by RNA-Seq, and fusion genes included *RPLP1-C19MC*, potentially deregulating the *C19MC* cluster, an imprinted locus containing microRNA genes reactivated by gene fusion in embryonal tumors with multilayered rosettes. Since the presumed cell of origin of MNTI is from the neural crest, we also compared gene expression with a dataset from human neural crest cells and identified 185 genes with significantly different expression. Consistent with the melanotic phenotype of the tumor, elevated expression of tyrosinase was observed. Other highly expressed genes encoded muscle proteins and modulators of the extracellular matrix. A derived MNTI cell line was sensitive to inhibitors of lysine demethylase, but not to compounds targeting other epigenetic regulators.

**Conclusions:**

In the absence of somatic copy number variations or mutations, the fully transformed phenotype of the MNTI may have arisen in infancy because of the combined effects of a germline *CDKN2A* mutation, tumor promoting somatic fusion genes and epigenetic deregulation. Very little is known about the etiology of MNTI and this report advances knowledge of these rare tumors by providing the first comprehensive genomic, transcriptomic and epigenetic characterization of a case.

**Electronic supplementary material:**

The online version of this article (doi:10.1186/s12885-016-2669-3) contains supplementary material, which is available to authorized users.

## Background

Melanotic neuroectodermal tumor of infancy (MNTI) presents as a painless, pigmented, rapidly expansile and lobulated lesion that primarily affects infants in their first year of life, with 80 % of patients being less than 6-months-old (extensively reviewed by Kruse-Lösler in 2006) [1]. Up to 92.8 % cases of MNTI affect the head and neck, mostly the maxilla (68–80 %), skull (10.8 %), mandible (6 %) and brain (4.3 %) [1]. There is a slight trend towards a greater incidence in males than females (ratio = 1.48) [1]. MNTI is generally considered to be benign, although the tumors grow rapidly, can be invasive and metastasize in 6.5 % of cases [2]. The treatment of choice for MNTI is radical surgery and a curative outcome is achieved in the majority of cases [2, 3]. Where surgery might be mutilating, chemotherapy has occasionally been successful, but it is generally considered that chemotherapy and radiotherapy are ineffective in controlling this disease [1, 4]. Post-operative recurrence rates for MNTI range from 10 to 60 % [3]. Late-returning disease following resection is rare, however, 39.3 % of all cases develop recurrence within 4 weeks of surgery and up to 71.4 % of cases recur within 4 months [1].

Microscopically, MNTI is described as a biphasic tumor consisting of small, round ‘neuroblast’-like cells with scant cytoplasm and larger, melanin-expressing, polygonal, ‘epithelial’-like cells in a desmoplastic stroma [5–7]. It is unclear which of these cell populations represent the main proliferative component of the tumor. Whereas immunohistochemistry indicates that expression of cell cycle proteins is restricted to the melanocytic cells [8], in cases where the MNTI has exhibited malignant behavior and metastasis, the dominant cell type has appeared neuroblastic [5, 6]. A neural crest origin for MNTI was proposed independently by Misugi et al*.* [9], on the basis of electron microscopic examination of a tumor, and Borello and Gorlin [10] who also observed that the high urinary levels of vanillylmandelic acid, the main end-stage metabolite of catecholamines, returned to normal in a patient after the tumor had been excised. Subsequent electron microscopy studies [7] have identified ultra-structural features, including characteristic melanin granules and modified tight-junctions that support the view that MNTI is derived from the neural crest. We consider this report to be of interest as it is, to the best of our knowledge, the first comprehensive genomic and transcriptomic characterization of an MNTI. The patient reported here is only the eighth case of MNTI presenting in an extremity, and the first reported in the fibula.

## Case presentation

**Fig. 1:**
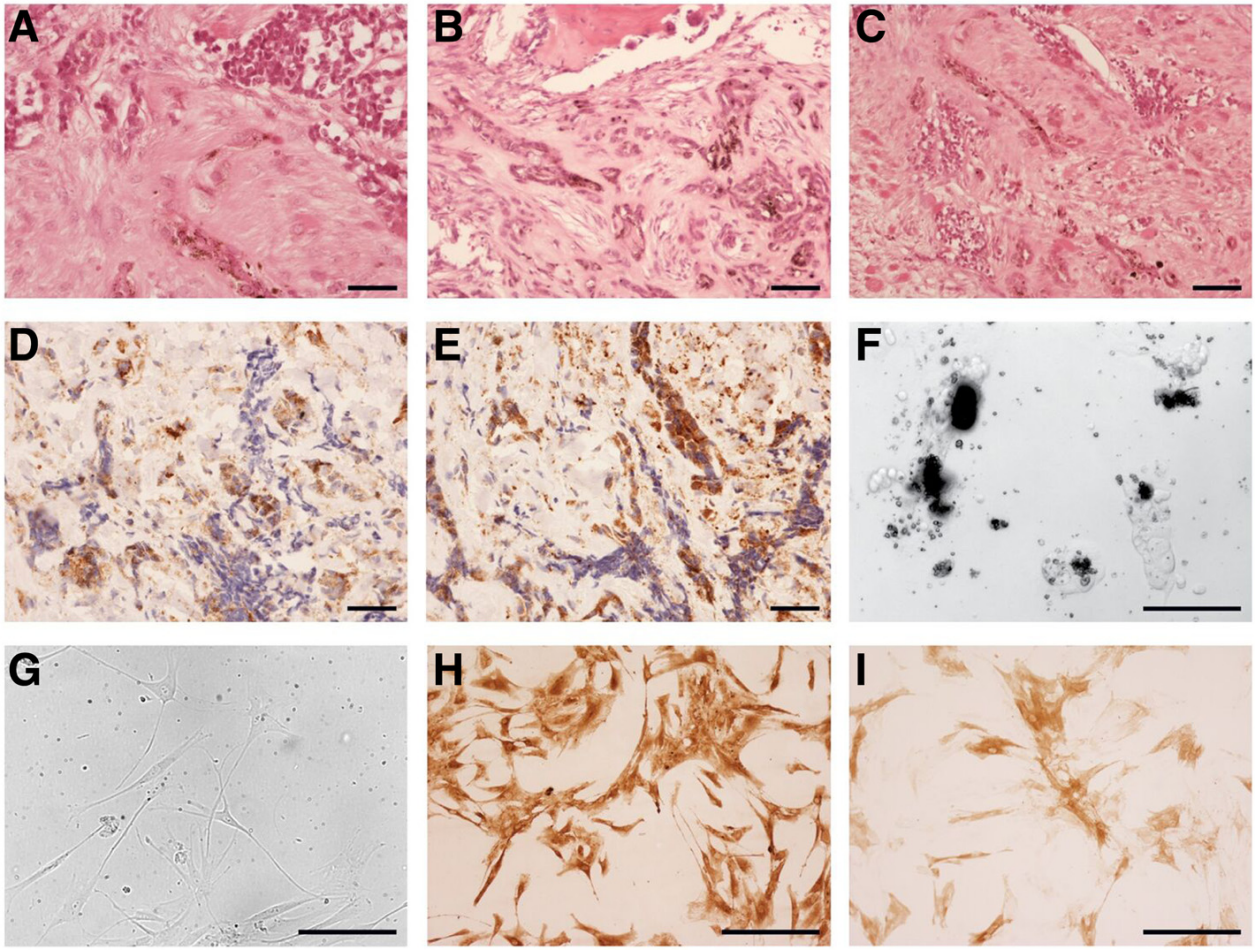
Pathological characterization of the MNTI tumor. The two main tumor cell types are readily apparent following haematoxylin and eosin-stained section from the tumor: (**a**) nests of small round cells with scant cytoplasm and hyperchromatic nuclei (top) and cords of polygonal, ‘epithelial-like’ cells containing speckles of melanin (bottom center), (**b**) Scattered pigment-containing tumor cells in bone, (**c**) MNTI tumor cells in soft tissue with residual muscle fibers evident. Tumor cells stain positive for CD99 (**d**) and HMB45 (**e**). **f** Bright field images of the cell line derived from the MNTI at passage 2 produced copious amounts of melanin causing cell clumps to appear black. **g** Phase contrast image of passage 6 of the cell line no longer synthesized melanin and the cells had adopted a more fibroblast-like morphology but remain positive for HMB45 (**h**) and NSE (**i**). Scale bars = 50 μm

A 2-month old female was referred to the Nuffield Orthopaedic Centre (Oxford) after her parents noticed a swelling on her left lower leg. A pigmented tumor measuring 5 × 2.5 × 2.5 cm was excised with wide margins. Upon histological examination, clumps and cords of tumor cells with scanty cytoplasm and large hyperchromatic or vesicular nuclei were observed. Some of the tumor cells also contained pigment of melanin (Fig. 1a, b, c). Immunohistochemistry showed that the tumor cells expressed vimentin, CD99 (Fig. 1d), HMB45 (Fig. 1e), NSE and cytokeratin (CK7+, CK20-). Nuclei stained for BAF47 and a high fraction of cells were proliferative identified with Ki-67. Some of the stromal cells stained for epithelial membrane antigen. There was also stromal staining for smooth muscle antigen and muscle actin. There was no specific staining for GFAP, myogenin, CD68, melan A, chromogranin, FABP4/aP2, CD117, podoplanin, alpha-fetoprotein, HCG, CD34, caldesmon, CD3, S100, CD45, desmin, CD20 and CD31. The tumor was present within the bone medulla and had spread through the cortex into covering muscle, fat and fibrous tissue. There were focal areas of tumor necrosis. Morphological features and immunohistochemistry were consistent with an MNTI. The patient is the subject of follow-up, and 3 years after surgery remains well with no recurrence.

We derived a cell line from the tumor that at low passage (<5) consisted of two distinct populations of adherent cells, one of which was highly melanotic (Fig. 1f). After 5 passages in culture, the densely pigmented cells were no longer apparent, and the culture consisted of fibroblasts which no longer produced melanin (Fig. 1g). Immunohistochemical analysis of the cells at passage 6, however, revealed a profile in keeping with the surgical resection specimen with strong staining for NSE and HMB45.

### Characterization of the MNTI genome

**Fig. 2:**
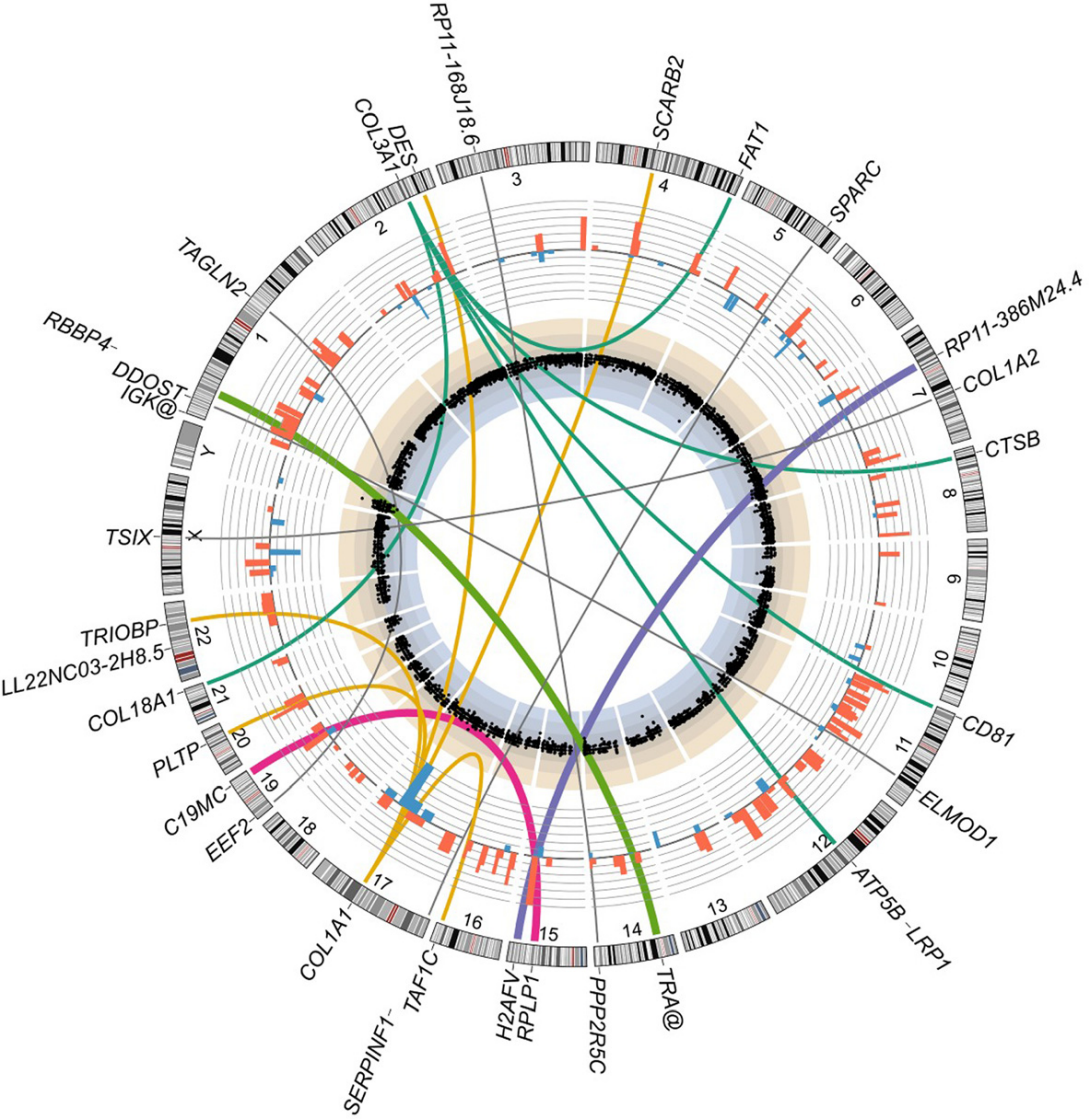
Genomic status of MNTI primary tumor. Circos plot of genomics data. Innermost track: Scatterplot of log_2_ (signal intensity ratios) for SNP array probes. Genomic DNA from the patient’s tumor and blood (reference signal) were hybridized to a custom 4 x 180 k SNP array containing probes for the whole-genome with a greater density for 1500 cancer-associated genes. There were no apparent copy number variations and the tumor was euploid. Blue and beige shaded sections represent ranges over which genomic losses or gains, respectively, would be expected to occur. Bar graph track: 185 genes are significantly (adjusted *P*-value ≤ 0.05) up-regulated (red) or down-regulated (blue) in the MNTI relative to their expression in an RNA-Seq dataset (GEO accession: GSE28875) for *in vitro*-differentiated human neural crest cells (hNCC). Bars represent log_2_ (FPKM + 0.01) fold-changes. Links: thin gray links are shown between partner genes for fusions where the reciprocal has not been detected, except for *COL3A1* and *COL1A1,* where rearrangements with multiple partner genes are predicted by the FusionCatcher analysis. Thicker links are shown for fusion genes where the reciprocal has been detected: *RPLP1-C19MC* (magenta), *H2AFV-RP11-386 M24.4* (purple) and *RBBP4-TRA@* (green)

As many pediatric small round cell tumors have either amplification of chromosomal regions containing proto-oncogenes or deletion of regions containing tumor suppressor genes, our expectation was that the MNTI would have copy number variations (CNV). By hybridizing genomic DNA from the MNTI and from the patient’s blood (the reference signal) to a human whole-genome SNP array (4 × 180 k design with a greater density of probes covering 1500 cancer-associated genes), the CNV data proved to be featureless (Fig. 2, innermost track), indicating that the MNTI was predominantly euploid.

Whole-exome sequencing on genomic DNA from both the MNTI and from the patient’s blood, aimed to identify somatic variants by subtracting the germline variants from the total variants detected in the MNTI. All of the somatic single nucleotide variants (SNVs) found in the tumor were synonymous. The patient had eight germline SNVs, which were predicted to be deleterious by any or all of the SIFT, PolyPhen or Condel algorithms (www.ensembl.org/info/docs/tools/vep/index.html), and four germline indels. All but one of the indels had prior annotation in dbSNP, and seven of the SNVs may be regarded as SNPs because they were annotated as such in either dbSNP or HGMD. Of most interest, was the SNV in exon 2 of *CDKN2A* (Chromosome 9:21971137, T to G) which causes mutation of aspartic acid to alanine at position 74 in p16^INK4A^ and is listed in COSMIC (COSM4163712) as a rare somatic mutation (Table 1). This variant is not a known founder mutation and, until now, has never been described in the germline. In the alternate reading frame of *CDKN2A*, the corresponding variant is synonymous causing a silent R88R mutation in p14^ARF^. No parental DNA was available to identify whether the *CDKN2A* (p16^INK4A^, D74A) mutation was a new mutation or inherited.

**Table 1:** Germline variants in the MNTI

Gene	Variant type	Chr	Position	Ref/Seq	Amino acid	Transcript	Read depth	Existing annotation (Allelic frequency)	Sift	Polyphen	Condel
*CDKN2A*	SNV	9	21970916	C/T	Ala/Thr	ENST00000304494.9	322	CM004869 (0.027)	deleterious	benign	neutral
*CDKN2A*	SNV	9	21971137	T/G	Asp/Ala	ENST00000304494.9	105	COSM4163712 (NA)	deleterious	probably_damaging	deleterious
*CHD1*	Indel	5	98192165	AGG/–	Pro/Unknown	ENST00000284049.7	NA	NA	NA	NA	NA

No tumor-specific non-synonymous mutations were identified and these variants were also detected in germline DNA extracted from the patient’s blood. A heterozygous T/G mutation on chr 9:21971137 generates a mutant allele of *CDKN2A* which encodes a p16^INK4A^ protein with substitution of aspartic acid for alanine at position 74. This change is predicted to be damaging or deleterious by the Sift, Polyphen and Condel algorithms and mutations of Asp74 in *CDKN2A* are documented in COSMIC as rare tumor variants. The other *CDKNA* mutation (Ala/Thr) is not predicted to be damaging to the function of p16^INK4A^. Common polymorphisms with a dbSNP annotation have been excluded

*SNV* single nucleotide variant, *NA* = not available

### Characterization of the MNTI transcriptome and detection of fusion genes

By RNA-Seq, we wished to identify differentially expressed genes and fusion genes that might be relevant as potential drivers of the tumorigenesis. As the tumor had been frozen, we were able to extract high quality RNA that was subjected to ultra-high depth (>200 million reads), paired-end RNA sequencing. Analysis with FusionCatcher [11] yielded 50 candidate fusion genes (Table 2 and Additional file 1: Table S1), indicating that the tumor had genomic instability. Intra-chromosomal events were required for 29 of the fusion genes and 23 of these were predicted to be read-through transcripts. Chromosomal translocations were involved in the formation of the remaining 21 fusion genes and for three of these (*H2AFV-RP11-386 M24.4*, *C19MC-RPLP1* and *RBBP4-TRA@*) reciprocal fusions were identified suggesting balanced translocation had occurred. The fusion of *RPLP1*, which encodes the large P1 ribosomal protein, to *C19MC*, the largest microRNA cluster in the human genome, located on chromosome 19q13.41, appears potentially functional. The ~100 kb cluster of 46 microRNA genes is imprinted, with expression largely confined to the first trimester of gestation [12]. Four collagen genes (*COL1A1*, *COL1A2*, *COL3A1* and *COL18A1*) were also re-arranged by chromosomal translocation in the MNTI, with *COL1A1* and *COL3A1* having multiple fusion partners (Fig. 2).

**Table 2:** Fusion genes (50) detected in the MNTI by FusionCatcher analysis of RNA-Seq data

Fusion gene	Location of 5’ partner	Location of 3’ partner	Cause of fusion
*RPLP1-C19MC*	15:69745292:+	19:54278860:+	Balanced chromosomal translocation
*C19MC-RPLP1*	19:54278886:+	15:69745266:+	Balanced chromosomal translocation (reciprocal fusion)
*RP11-386 M24.4-H2AFV*	15:93276847:-	7:44873962:-	Balanced chromosomal translocation
*H2AFV-RP11-386 M24.4*	7:44875191:-	15:93277223:-	Balanced chromosomal translocation (reciprocal fusion)
*TRA@-RBBP4*	14:22032433:+	1:33133924:+	Balanced chromosomal translocation
*RBBP4-TRA@*	1:33146113:+	14:22034317:+	Balanced chromosomal translocation (reciprocal fusion)
*COL1A1-DES*	17:48261671:-	2:220290692:+	Chromosomal translocation
*COL1A1-SCARB2*	17:48262192:-	4:77109675:-	Chromosomal translocation
*COL1A1-PLTP*	17:48264070:-	20:44537829:-	Chromosomal translocation
*COL1A1-TRIOBP*	17:48264212:-	22:38126504:+	Chromosomal translocation
*COL3A1-COL18A1*	2:189851865:+	21:46925141:+	Chromosomal translocation
*COL3A1-FAT1*	2:189871155:+	4:187561585:-	Chromosomal translocation
*COL3A1-CD81*	2:189871176:+	11:2411930:+	Chromosomal translocation
*COL3A1-ATP5B*	2:189873714:+	12:57037364:-	Chromosomal translocation
*COL3A1-LRP1*	2:189876968:+	12:57597641:+	Chromosomal translocation
*CTSB-COL3A1*	8:11703238:-	2:189873743:+	Chromosomal translocation
*DDOST-ELMOD1*	1:20978357:-	11:107525369:+	Chromosomal translocation
*EEF2-TAGLN2*	19:3982389:-	1:159888278:-	Chromosomal translocation
*IGK@-LL22NC03-2H8.5*	2:90296180:+	22:22640524:-	Chromosomal translocation
*PINK1-AS-ELMOD1*	1:20978357:-	11:107525369:+	Chromosomal translocation
*PPP2R5C-RP11-168 J18.6*	14:102375968:+	3:52408668:+	Chromosomal translocation
*SERPINF1-SPARC*	17:1680627:+	5:151044730:-	Chromosomal translocation
*TAF1C-COL1A1*	16:84218540:-	17:48268811:-	Chromosomal translocation
*TSIX-COL1A2*	X:73020413:+	7:94060360:+	Chromosomal translocation
*COL3A1-CHPF*	2:189860508:+	2:220404337:-	Intra-chromosomal deletion
*FBXL18-TNRC18*	7:5530862:-	7:5434226:-	Intra-chromosomal deletion
*LSP1-TNNT3*	11:1908806:+	11:1944087:+	Intra-chromosomal deletion
*SMC5-AS1-MAMDC2-AS1*	9:72831202:-	9:72703410:-	Intra-chromosomal deletion
*TFDP2-XRN1*	3:141820577:-	3:142084208:-	Intra-chromosomal deletion
*TPST1-GS1-124 K5.4*	7:65751696:+	7:65960242:+	Intra-chromosomal deletion
*ARMC9-AC017104.6*	2:232234836:+	2:232258192:+	Read-through transcript
*BCKDK-KAT8*	16:31123348:+	16:31128784:+	Read-through transcript
*C17ORF107-GP1BA*	17:4902763:+	17:4934342:+	Read-through transcript
*C1GALT1-AC005532.5*	7:7283381:+	7:7317216:+	Read-through transcript
*CD4-P3H3*	12:6925379:+	12:6938651:+	Read-through transcript
*CHD4-NOP2*	12:6680035:-	12:6677088:-	Read-through transcript
*EIF3K-ACTN4*	19:39116742:+	19:39191240:+	Read-through transcript
*FAM89B-EHBP1L1*	11:65341233:+	11:65346549:+	Read-through transcript
*GPR141-NME8*	7:37725186:+	7:37896876:+	Read-through transcript
*GPX7-FAM159A*	1:53072617:+	1:53108535:+	Read-through transcript
*HEPHL1-PANX1*	11:93800909:+	11:93862494:+	Read-through transcript
*LMO1-RIC3*	11:8248522:-	11:8174970:-	Read-through transcript
*LMO7-AS1-COMMD6*	13:76209965:-	13:76123565:-	Read-through transcript
*NPTXR-DNAL4*	22:39219088:-	22:39177014:-	Read-through transcript
*RFPL1-NEFH*	22:29838118:+	22:29876241:+	Read-through transcript
*SIX3-AC012354.6*	2:45171866:+	2:45193557:+	Read-through transcript
*SLC25A43-SLC25A5*	X:118544325:+	X:118602415:+	Read-through transcript
*SSSCA1-EHBP1L1*	11:65341233:+	11:65346549:+	Read-through transcript
*TBC1D23-NIT2*	3:100039815:+	3:100057931:+	Read-through transcript
*TMEM101-PYY*	17:42100940:-	17:42043900:-	Read-through transcript
*VPS45-PLEKHO1*	1:150082742:+	1:150123102:+	Read-through transcript
*ZCWPW2-LINC00693*	3:28562607:+	3:28616561:+	Read-through transcript
*ZNF836-ZNF616*	19:52663718:-	19:52633841:-	Read-through transcript

Location of fusion points is given as: chromosome : genomic coordinates : strand. Where FusionCatcher has returned multiple fusion point coordinates, the location with the most unique spanning reads is listed. Reciprocal fusions, indicative of balanced chromosomal translocations, were detected for three fusion genes (*C19MC-RPLP1, H2AFV-RP11-386 M24* and *RBBP4-TRA@*). Two fusion genes reported by FusionCatcher as being ‘probable false positives’ have been excluded

Since the presumed cell of origin of MNTI is from the human neural crest (7,9,10), we also asked how the transcriptional profile of our patient’s tumor might differ from that of human neural crest cells (hNCC). Rada-Iglesias et al. [13] developed an *in vitro* model which recapitulates neural crest formation during gestation by inducing differentiation of human embryonic stem cells, first into neuroectodermal spheres, and then into migratory hNCC. We compared our RNA-Seq expression data from the tumor with the single RNA-Seq dataset obtained from the differentiated hNCC (GEO acession: GSE28875). The transcriptional profiles were highly similar, in keeping with an hNCC origin for MNTI, with no significant differences in expression for 24146 transcripts out of 24331. We identified just 185 genes with expression that was significantly different (adjusted *P*-value ≤ 0.05, Fig. 2, bar graph track). The 25 genes showing the greatest differences in expression (Table 3) encode proteins with diverse functions including: ribosomal proteins (*RPS17*), serum transport proteins (*TTR*), transcription factors (*POU3F3*, *TFAP2B*, *SP8*) and extracellular matrix proteins (*SPON2*, *DPT*). Amongst them is a subgroup of genes that encode components of muscle (*TNNT3*, *MYL1*, *MYL2*, *TNNI2*), consistent with the finding of strong staining of stromal cells for smooth muscle actin and muscle actin, suggesting that the MNTI has undergone myogenic differentiation. Of note, *TYR,* which encodes tyrosinase, the enzyme that catalyzes the initial steps in the biosynthesis of melanin from tyrosine, was highly expressed in the MNTI, in keeping with its melanotic phenotype, but was not expressed in hNCC.

**Table 3:** The 25 most differentially expressed genes in the MNTI relative to the hNCC dataset

Gene	Description	Chromosome	Expression (FPKM)	
			MNTI	hNCC
RPS17	Ribosomal protein S17	15	1616.3	0.0
TTR	Transthyretin	18	532.5	2.6
THBS2	Thrombospondin 2	6	298.3	0.5
TNNT3	Troponin T type 3 (skeletal, fast)	11	266.4	0.0
BPIFB4	BPI fold containing family B, member 4	20	262.5	0.0
TMEM176A	Transmembrane protein 176A	7	172.9	0.0
SPON2	Spondin 2, extracellular matrix protein	4	169.2	2.0
HLA-DRA	Major histocompatibility complex, class II, DR alpha	6	162.7	0.0
TYR	Tyrosinase	11	144.2	0.0
MYL1	Myosin, light chain 1, alkali; skeletal, fast	2	141.2	0.0
FDCSP	Follicular dendritic cell secreted protein	4	117.7	0.0
MYL2	Myosin, light chain 2, regulatory, cardiac, slow	12	117.4	0.0
LRRC15	Leucine rich repeat containing 15	3	105.6	0.0
IL32	Interleukin 32	16	105.6	0.0
DPT	Dermatopontin	1	99.9	0.0
LYPD2	LY6/PLAUR domain containing 2	8	73.3	0.0
TNNI2	Troponin I type 2 (skeletal, fast)	11	71.2	0.0
MEG3	Maternally expressed 3 (non-protein coding)	14	57.9	0.6
MIR770	microRNA 770	14	57.9	0.6
MT1E	Metallothionein 1E	16	46.2	0.0
TYROBP	TYRO protein tyrosine kinase binding protein	19	43.2	0.0
SLC1A7	Solute carrier family 1 (glutamate transporter), member 7	1	42.5	0.0
POU3F3	POU class 3 homeobox 3	2	0.0	54.3
MTRNR2L10	MT-RNR2-like 10	X	0.0	89.6
MIR4737	microRNA 4737	17	0.0	1873.8

*FPKM* fragments per kilobase per million mapped reads, *hNCC* human neural crest cells

The microRNA (miR) gene *MIR4737*, was the most highly expressed gene in hNCC relative to the MNTI, in which it was not expressed. Down-regulation of expression of *MIR4737* in the MNTI implies that the target genes for this miR will be up-regulated. From TargetScan (www.targetscan.org), we identified 465 potential targets of hsa-miR-4737, of which only two, *EDN2* and *MYOD1*, were significantly upregulated in the MNTI. The loss of repression of *MYOD1*, is also consistent with myogenic differentiation and the expression of muscle component genes in the MNTI.

In order to gain insight into how genes differentially expressed in the MNTI, relative to hNCC, might be functioning in a coordinated manner, we carried out a gene set enrichment analysis (GSEA). A total of 28 gene sets were upregulated in the MNTI (data not shown), of which the set having the highest normalized enrichment score was from genes that are enriched in invasive ductal breast carcinoma [14]. The leading edge set of genes from the MNTI enriched in this set comprised: *THBS2*, *SPON2*, *HLA-DRA*, *LRRC15*, *TYROBP*, *MMP13*, *LY96*, *C1QB* and *C1QA*. The coordinated expression of these genes is suggestive of an epithelial to mesenchymal transition (EMT) mediated by the transcriptional regulator Twist1 [14], even though *TWIST1* expression was not significantly different in the MNTI from its expression in hNCC.

### Sensitivity of a cell line derived from the MNTI to inhibitors of Cdk4/Cdk6 and lysine demethylase

**Fig. 3:**
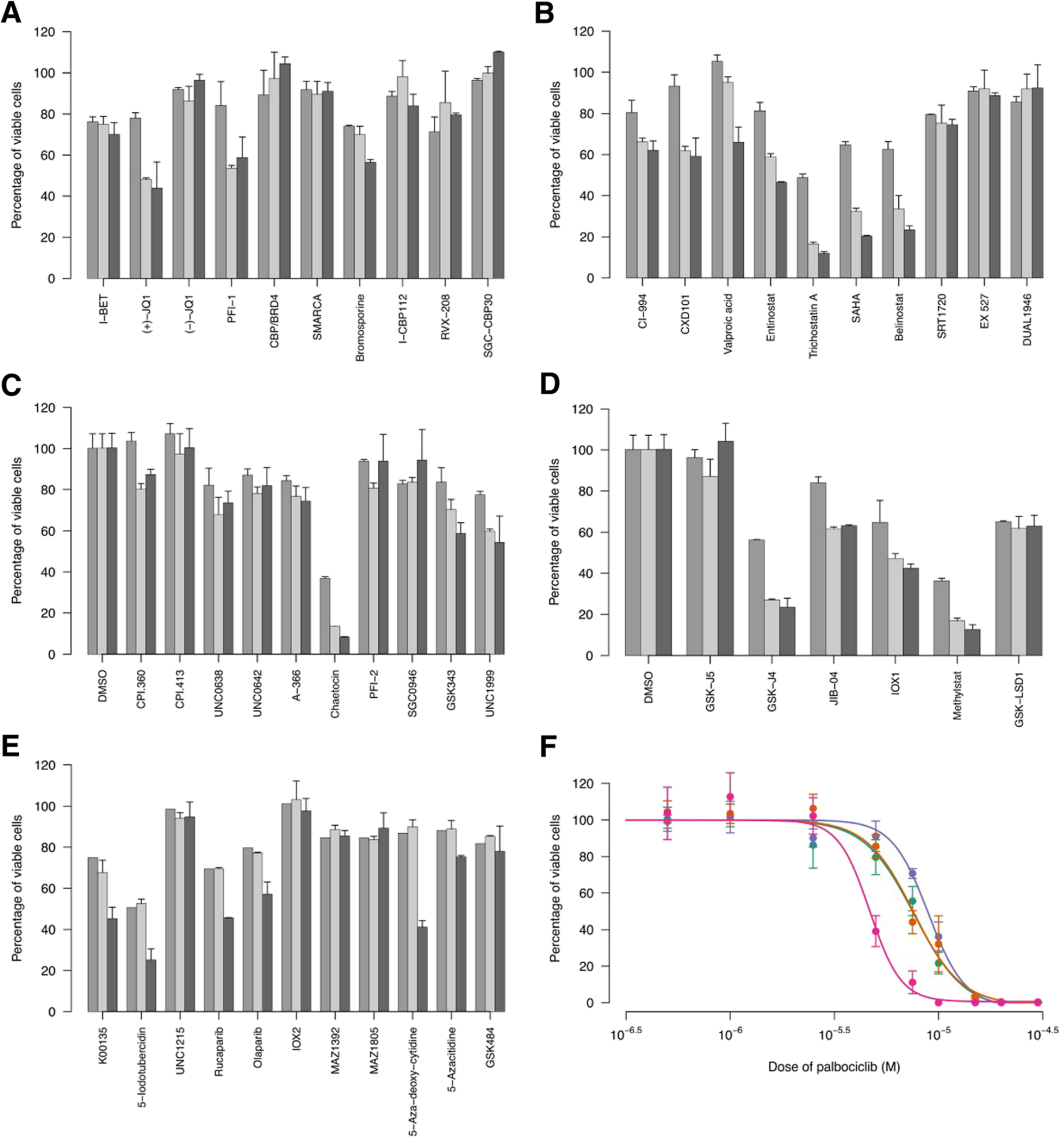
Responses of MNTI cell line to epigenetic inhibition and a CDK4/CDK6 inhibitor. MNTI cells were treated with bromodomain inhibitors (**a**), histone deacetylase inhibitors (**b**), histone methyltransferase inhibitors (**c**), lysine demethylase inhibitors (**d**) or miscellaneous inhibitors (**e**). Cell viability was reduced in response to a range of small molecule inhibitors of lysine demethylases (**d**) but was unaffected by inhibitors of other epigenetic mechanisms. Results are normalized to the vehicle control and represent the mean ± s.d. of either 3 biological replicates (day 3, mid gray) or 2 biological replicates (days 7, light gray and 10, dark gray). **f** Log dose–response curves for CD4/CDK6 inhibitor palbociclib-treated MNTI cells (, magenta) and Ewing sarcoma cell lines: CHP-100 (, purple), A673 (, teal) and SK-N-MC (, orange). Cells were treated with doses of palbociclib or vehicle control (DMSO) for 72 h and those remaining viable were assayed using the MTS reagent. Percentage of viable cells was calculated by normalizing absorbance readings taken at 490 nm. Data are mean ± s.e.m. and representative curves from one of three independent experiments are shown. Inhibitors in (**e**) targeted: kinases (K00135, 5-iodotubercidin), a methyl-lysine reader (UNC1215), PARP (rucaparabib, olaparib), PHD2 (IOX2), tRNA synthetase (MAZ1392, MAZ1805), DNA methyltransferase (5-aza-deoxy-cytidine, 5-azacitidine) and arginine deiminase (GSK484 low and high doses shown)

We reasoned that the potential loss of regulation of Cdk4/Cdk6 due to mutation of an allele of *CDKN2A* would be expected to make cells of the MNTI more susceptible to cytotoxicity induced by Cdk4/Cdk6 inhibition. To test this, we treated the cell line derived from the MNTI with palbociclib, a specific small molecule inhibitor of Cdk4/Cdk6 [15]. For comparison, we also treated Ewing sarcoma cell lines with palbociclib that were wild-type (SK-N-MC) or had hemizygous deletion (CHP-100) or were nullizygous (A673) for *CDKN2A*, confirmed by SNP arrayCGH in our laboratory (results not shown). Although not statistically significant, differences in sensitivity were observed (Fig. 3f), with the MNTI cells proving to be the most sensitive to palbociclib (mean IC_50_ ± s.d., 6.01 ± 2.01 μM) and Ewing cell lines with *CDKN2A* deletions (CHP-100 and A673) being the least sensitive (mean IC_50_ ± s.d., 8.03 ± 0.84 and 7.92 ± 1.27 μM, respectively; MNTI IC_50_ vs. CHP-100 IC_50_, *P* = 0.4, Mann–Whitney test).

Since we had been unable to detect any unequivocal driver mutations, previously described oncogenic fusion genes or CNV that might account for tumorigenesis of the MNTI, we hypothesized that epigenetic mechanisms might be involved. Furthermore, we observed variegation upon deriving the MNTI cell line, with a population of differentiated, melanotic cells co-existing with a less differentiated population at early passages (Fig. 1f). The differentiated cells did not persist into later passages (Fig. 1g), which suggested a phenotypic switch. Accordingly, we tested a selection of 47 small molecule inhibitors targeted against a broad range of proteins involved in epigenetic processes to investigate the importance of epigenetic alterations on the viability and phenotype of the MNTI cell line in culture (Fig. 3). Our rationale for testing this panel was that it would permit candidate enzymes to be identified that were either required for maintaining growth of the tumor or that were responsible for the phenotypic switch to a less differentiated state. Viability was reduced when cells were treated with compounds targeting lysine demethylase enzymes (Fig. 3d). Enzymes affected included: the 2-oxygluterate (2-OG) oxygenase family, inhibited by IOX1, the Jumonji C (JmjC) domain-containing superfamily, inhibited by JIB-04 and Methylstat, and JARID1B/KDM5B, UTX/KDM6A and lysine demethylase 6B, which were all targets of GSK-J4. GSK-J5, a less active isomer of GSK-J4 had little effect. In contrast, bromodomain inhibitors (Fig. 3a), histone deacetylase inhibitors (Fig. 3b) and methyltransferase inhibitors (Fig. 3c) did not cause cell death, with the exception of chaetocin, which also inhibits thioredoxin reductase [16]. The sensitivity of the MNTI cell line to compounds targeting different classes of epigenetic regulators (Fig. 3e), though variable, was less than for inhibitors of lysine demethylase enzymes. Although none of these results achieved statistical significance (pairwise Wilcoxon rank sum tests with Holm correction, all *P* > 0.05) they do point to a trend. Overall, our data suggest that epigenetic mechanisms and, in particular, the balance between methylation and demethylation of histone lysines are required for maintaining the viability of tumor cells in the MNTI.

## Conclusions

Presentation of MNTI in the extremities is rare, with only 7 previous cases having been reported, and so this case may not be representative of all reported cases. In 6 cases the lower limb was affected, with the tumor originating in the femur [4, 17–19] or in soft tissues [20, 21]. There is a single report of a case of MNTI in the upper limb [22]. Femoral MNTIs appear to exhibit malignant features and had a poor clinical outcome. In the case described by Johnson et al*.* in 1983, an 18-month-old girl had an aggressive MNTI in her left femur which metastasized to the pelvis within 2 months [4]. In the cases reported by Elli et al*.* [17] and Choi et al*.* [18], the tumors were both large and surgery was considered too mutilating. The first patient, a 3-month-old female infant, was lost to follow-up after the parents refused chemotherapy [17], while the second, a 5-month-old male, died of heart failure owing to the cardiotoxicity of the chemotherapy [18]. The femoral MNTI reported by Rekhi et al*.* was also large but was successfully resected and the patient, a 12-month-old male, was the subject of follow-up at the time of publication [19].

Despite such case reports, attempts at unbiased genetic characterization of MNTI tumors have not been reported. We exploited massively parallel next generation sequencing technologies to perform an exhaustive molecular genomic and transcriptomic survey of an MNTI. Whole-genome array-CGH data revealed the patient’s tumor to be euploid, suggesting that CNV are less likely to be involved in the etiology of MNTI in this case. We detected a heterozygous mutated *CDKN2A* (p16^INK4A^, D74A) allele, but not LOH of *CDKN2A* and transcripts from both alleles were expressed at levels comparable to those in hNCC, indicating the mutation may exhibit haploinsufficiency (data not shown). The absence of CNV is perhaps not surprising, given that they appear to occur less frequently in other small round cell tumors in childhood. Moreover, karyotypic analysis by Khoddami et al*.* [23] tested three MNTI for molecular lesions associated with other pediatric small cell tumors: *MYC* amplification, deletion of 1p (both of which are features of neuroblastoma), and chromosomal translocations (t(11;22)(q24;q12), t(11;22)(p13;q12), responsible for Ewing sarcoma and desmoplastic small round cell tumor, respectively. None of these abnormalities were detected.

Few cell lines have been derived from MNTI, of which the best characterized histologically and cytogenetically are the 3 MNTI lines described by Metwaly et al. [24]. These lines were hypotriploid, with a modal chromosome number of 65, and had multiple chromosomal abnormalities and CNV [24]. It is possible that the MNTI lines were heterogeneous, as the karyotypes and chromosomal abnormalities differed in the 3 clones despite being derived from the same original maxillary tumor. Although we did not specifically test ploidy in the derived cell line, our data suggest that either CNV or aneuploidy are not essential for MNTI tumorigenesis *in vivo*. The differentiation of the MNTI cell line was towards a melanotic appearance at initial early passage, but ceased to produce melanin as it was propagated in culture despite continued expression of HMB45. Our findings contrast with those of Metwaly et al*.*, who noted that their derived MNT1/2/3 cell lines did not produce melanin and were negative for HMB45 staining [24]. Partial melanocytic differentiation can be induced by treating the cells with endothelin-3 and vitamin D_3_, which restored HMB45 expression, but not melanin production [24]. In addition, we did not observe the structural aberrations reported for these cell lines, for example, der(9)t(9;13)(p13;q12)add(9)(q34) and der(13;21)(q10;q10) were present in all 3 of the MNTI lines, and a der(19)t(11;19)(q13;p13) was detected in 2 of the cell lines [24].

Our most notable observation was the detection of a heterozygous, germline missense mutation in *CDKN2A*, D74A, in this case which is predicted to be deleterious by the Variant Effect Predictor (release 77, Ensembl). Germline mutations in *CDKN2A* are responsible for familial melanoma syndromes, such as the intensively studied p16-Leiden truncation mutation which causes familial atypical multiple mole-melanoma susceptibility [25]. Since *CDKN2A* mutations drive the proliferation of melanocytes in familial melanoma and, as the large melanin-expressing, epithelial-like cells in MNTI are, most likely, immature melanocytes, we do not consider the D74A to be simply a passenger mutation but infer a role for it in contributing to tumor growth in this patient. Little is known concerning rates of LOH in familial melanoma and whether this is necessary for the disease phenotype. In one immunohistochemical study of p16 expression in 98 familial melanoma patients [26], a single patient was found to be hemizygous for p16 suggesting that LOH, at least in early stage tumors, is comparatively rare. Also, *CDKN2A* D74 mutations are infrequent and have never been described in melanoma, either familial or sporadic, although a single report exists of a somatic *CDKN2A* D74E mutation (COSM13768) in a sporadic dysplastic nevus [27]. Regardless of its role in tumorigenesis of the MNTI, it is probable that the germline D74A *CDKN2A* mutation may also predispose patients to an increased risk of developing other cancers later in life. Inferences may be drawn from the analyses of cancer risk from previous large-scale studies. For individuals carrying germline mutations in *CDKN2A*, the risk of developing melanoma before the age of 80 has been estimated to be 67 % [28]. In families carrying the p16-Leiden mutation, the relative risk of developing cancers other than melanoma is 4.4 [29] with a cumulative risk of developing pancreatic cancer before the age of 75 of 17 % [30].

In terms of the functional effect of the variant in *CDKN2A* (p16^INK4A^, D74A) on protein function, it is likely that this variant is significant, as mutation of aspartic acid 74, to asparagine has been identified in bladder carcinoma [31] and esophageal squamous cell carcinoma [32]. Mutation of D74 to alanine was subsequently detected in a non-small cell lung carcinoma [33], with five somatic, non-synonymous *CDKN2A* D74 mutations listed in COSMIC for twelve samples: D74Y (COSM12509, larynx, lung, esophagus, gallbladder), D74N (COSM13474, thyroid, esophagus), D74V (COSM13546, larynx, bile duct), D74A (COSM4163712, thyroid) and D74E (COSM13768, skin). Although D74A has not been studied *in vitro,* a homologous mutation, D74N, was reported as being one of four p16^INK4A^ mutations which caused significant disruption of the protein’s secondary structure, assessed by circular dichroism spectroscopy, with the misfolding making the protein more susceptible to proteolysis than wild-type p16 [34]. The functional consequences of the D74N mutation were investigated by Yarbrough et al*.* [35], who found that although the mutant protein bound CDK4 and CDK6 with activity comparable to wild-type p16, by affinity co-precipitation, it was defective in its ability to inhibit CDK6 kinase activity in an *in vitro* assay. In addition, when ectopically expressed at low levels in U2OS cells, the D74N mutant was unable to induce cell cycle arrest in G_1_ [35]. The aspartic acid residue at position 74 of p16 is evolutionarily conserved and occurs at the C-terminal of the protein’s second ankyrin repeat [35]. We would predict that the loss of function in the p16 expressed from the mutant allele in our patient would be more severe, as mutation of aspartic acid to asparagine is a conservative substitution, whereas mutation of aspartic acid to alanine involves loss of an acidic carboxyl side chain and replacement of a hydrophilic residue with a hydrophobic one. Despite identification of somatic D74A, this mutation has not been previously observed in the germline.

Although euploid, the genome of the MNTI appeared highly unstable as evidenced by multiple chromosomal rearrangements and fusion transcripts. We detected three fusion genes with their reciprocals, which increases our confidence that they are not false positives but are most likely products of balanced chromosomal translocations. The *C19MC-RPLP1* fusion may be relevant as fusions of *C19MC* with *TTHY1* are found in embryonal tumors with multilayered rosettes (ETMRs), where they are believed to be responsible for the re-engagement of a neurodevelopmental program that causes tumorigenesis [12]. Deregulation and amplification of *C19MC* by fusion with *TTHY1* is known to drive the expression of microRNAs that suppress expression of retinoblastoma-like 2, *RBL2*. Suppression of *RBL2*, in turn, prevents full methylation and silencing of promoter 1B of a DNA methyltransferase, *DNMT3B*, leading to the excessive transcription of its fetal brain-specific isoform containing alternative exon 1B. It is unlikely that exactly the same mechanism is active in the MNTI, as we failed to detect either abnormally high *DNMT3B* expression or suppression of *RBL2* expression (data not shown). It is possible, however, that fusion of *RPLP1* to *C19MC* leads to the deregulation of different, and potentially tumorigenic, microRNAs within the cluster that have different targets. To test this hypothesis, miRNA-Seq using a small RNA library would be required to quantify the expression of mature microRNA species from *C19MC*.

The functional consequences of the other reciprocal fusions in the MNTI, *RBBP4-TRA@* and *H2AFV-RP11-386 M24.4,* are difficult to predict but both involve epigenetic regulators. The protein product of *RBBP4* is retinoblastoma binding protein 4, a component of histone deacetylase complexes, which has been implicated in the transcriptional repression of E2F-responsive genes via direct binding to Rb [36]. Fusion with *TRA@*, the T-cell receptor alpha locus, implies a mechanism of overexpression similar to the inappropriate activation of the various transcription factor partner genes in T-precursor Acute Lymphoblastic Leukemia (ALL). *RBBP4*, however, has never been described as a partner gene in ALL and we found its expression to be lower, rather than higher, in the MNTI relative to the hNCC dataset. The *H2AFV-RP11-386 M24.4* fusion involves *H2AFV* (also known as *H2A.Z-2*), which encodes a member of the H2A family of histone proteins, and the pseudogene *RP11-386 M24.4*. An in-frame, exonic breakpoint is involved which would replace *H2AFV* coding sequences with those from the pseudogene. Since there are H2A transcript isoforms, however, and redundancy, this loss of function may be compensated by another H2A family member, such as *H2A.Z-1*. The reciprocal fusion, *RP11-386 M24.4-H2AFV*, will not be translated as *RP11-386 M24.4* is a pseudogene. With respect to the other fusions involving collagen genes, *COL1A1* encodes two α1 chains of type I collagen, the most abundant form in healthy connective tissues. The α1 chains form a heterotrimer with the α2 chain encoded by *COL1A2* [37]. Expression of *COL1A2* is frequently silenced by hypermethylation of its promoter in colorectal cancer, cancer cell lines [38] and in melanoma [37], suggesting a potential role as a tumor suppressor. Type I collagen is the major fibrillary component of stroma in solid tumors and may be produced either by the carcinoma cells themselves or by stromal fibroblasts [38].

Since multiple lines of evidence point to MNTI having a neural crest origin [1], it was of interest to profile gene expression in our patient’s tumor relative to untransformed hNCC. We required a comparator to assess changes in gene expression in our RNA-Seq data from baseline values. This was provided by an RNA-Seq dataset from an *in vitro* model of human neural crest development [13]. The comparison yielded a signature of 185 genes with significantly altered gene expression. As evidence of proof of concept, we readily identified tyrosinase, *TYR*, as being highly expressed in the MNTI, consistent with the excessive, pathophysiological biosynthesis of melanin. We did not detect strong expression of melanotransferrin (*MFI2*, CD228, MAP97) mRNA, which has been reported for an MNTI [39] or significantly elevated expression of MITF, microphthalmia-associated transcription factor, the master transcriptional regulator of melanogenesis. The gene most highly expressed in hNCC and silent in the MNTI was *MIR4737*. Of the putative hsa-miR-4737 targets, only *EDN2* and *MYOD1*, were significantly upregulated in the MNTI. Our GSEA results suggest that the pattern of expressed genes in the MNTI has features in common with a dataset for invasive ductal breast carcinoma [14]. *LRRC15* (to single out a gene from the leading edge set) encodes a leucine-rich transmembrane protein which, in normal tissue, is only expressed in the invasive cytotrophoblast layer of the placenta [40]. Considering the small round cell component of MNTI, it is noteworthy that *LRRC15* was first identified as being the transcriptional target of the *EWSR1-WT1(+KTS)* fusion gene in desmoplastic small round cell tumor [40].

In summary, our data delineate a potentially complex, multifactorial picture of the tumorigenic mechanisms likely to be responsible for MNTI. Our main findings are a germline mutation in *CDKN2A*, liable to pre-dispose to tumor formation, that the tumor was euploid, but expressed fusion genes caused by chromosomal translocations, and that there were differences in the expression of a limited number of genes, relative to the background of the neural crest, which may be key to the tumor phenotype. The MNTI signature contains genes associated with both melanocytic and myogenic differentiation, as well as genes present in invasive ductal breast carcinoma signatures. In the absence of CNV to account for altered gene expression, epigenetic mechanisms are implicated in the repression or de-repression of genes that cause the deviation from the normal physiology of the neural crest. The results of treating the MNTI cell line with a panel of inhibitors of epigenetic regulators are consistent with this and point to the involvement of altered methylation of histone lysines in maintenance of the disease phenotype. In addition, the fusion of *RPLP1* with *C19MC* suggests a microRNA-mediated epigenetic effect may be present which is analogous to, but not identical with, that caused by a *TTHY1-C19MC* fusion.

## Methods

### Tumor processing

A tumor measuring 5 × 2.5 × 2.5 cm was excised *en bloc* from the patient’s fibula. The tumor was snap frozen in liquid nitrogen and stored at −80 °C. Informed consent was obtained from the patient’s parents for the use of the tumor in research, in accordance with the ethics of the Oxford University Hospitals NHS Trust (REC C 09/H0606/11) and under the terms of the Human Tissue Act (HTA License 12217).

### RNA and DNA extraction, quality control and library preparation

Total RNA was extracted using an RNeasy Fibrous Tissue kit (Qiagen, Manchester, UK) from a 12.5 mg piece of the tumor, homogenized with a hand-held TissueRuptor homogenizer (Qiagen). Genomic DNA was extracted using a DNeasy Blood and Tissue kit (Qiagen) from a 19.1 mg piece of the tumor, homogenized with a TissueRuptor and digested overnight with proteinase K (Qiagen). Genomic DNA extracted from the patient’s blood was obtained from the Children’s Cancer and Leukaemia Group Biobank, Newcastle. For whole-exome sequencing, targets were captured using SureSelect (Agilent, Stockport, UK). Samples were prepared and enriched according to the manufacturer’s instructions. The concentration of each library was determined using a QPCR NGS Library Quantification Kit (G4880A, Agilent). Samples were pooled prior to sequencing with each sample at a final concentration of 10 nM.

### SNP arrayCGH, whole-exome sequencing and RNA-Seq

Aliquots of genomic DNA from the tumor and blood were hybridized to a Cytosure Cancer + small nucleotide polymorphism (SNP) array (Oxford Gene Technology, Begbroke, UK) and read on a SureScan scanner (Agilent). The array was a 4 × 180 k design, comprising a whole genome backbone with a greater density of probes for 1500 cancer-associated genes. Whole-exome sequencing was done on a MiSeq using TruSeq version 3 chemistry to generate 2 × 150 base reads (Illumina, Chesterford, UK). Paired-end RNA-Seq reads were acquired on a HiSeq 2500 (Illumina).

### Genomic and transcriptomic analysis

ArrayCGH data were analyzed using CytoSure Interpret software, version 4.3.2 (Oxford Gene Technology). For whole-exome sequencing, reads from Fastq files were mapped to the reference human genome (hg19/b37) with the Burrows-Wheeler Aligner (BWA) package, version 0.6.2. Local realignment of the mapped reads around potential insertion/deletion (indel) sites was carried out with the Genome Analysis Tool Kit, version 1.6 (GATK, Broad Institute). Duplicate reads were marked using Picard, version 1.89 (Broad Institute). Additional BAM file manipulations were performed with Samtools 0.1.18. Base quality scores were recalibrated using GATK’s covariance recalibration. SNP variants were called using VarScan2 and Indel variants were called using GATK SomaticIndelDetector. SNP novelty was determined against dbSNP release 135. Deleterious SNPs were identified using the Variant Effect Predictor, release 77 (Ensembl). Putative fusion genes were identified from RNA-Seq data using FusionCatcher, version 0.99.4c [11]. For analysis of transcript abundance from RNA-Seq data, analyses were carried out using the software tools in the Tuxedo pipeline [41]. Paired-end reads from Fastq files were aligned to the human genome (hg19/b37) with the TopHat-Bowtie2 aligner, versions 2.0.13 and 2.2.5, respectively, and expression of transcripts was quantified with Cufflinks, version 2.2.1, as fragments per kilobase per million mapped reads (FPKM). For statistical analysis of differential expression in RNA-Seq data, the getSig function of CummeRbund was used, with the default alpha of 0.05, to access genes with significantly different expression according to adjusted *P*-values calculated by Cufflinks [42].

### Derivation of MNTI cell line

A section of tumor approximately 0.5 cm^3^ was cut from the surgical resection specimen and was homgenized by hand prior to overnight digestion with Dispase II (Roche, Burgess Hill, UK). Cells were cultured in alpha MEM (Lonza, Slough, UK) with 20 % heat inactivated fetal calf serum, 4 mM glutamine, 100 units/mL penicillin and 100 μg/mL streptomycin in a humidified incubator at 37 °C and 5 % CO_2_. After approximately 2 weeks with no visible proliferation, the cell number began to increase. Cells were passaged at a ratio of 1:5 when they reached 80 % confluence using trypsin-EDTA (Sigma, Poole, UK). Immunohistochemical analysis of the cells at passage 6 was performed to characterize the identity of the proliferating cells.

### Screen of small molecule inhibitors of epigenetic regulators for compounds that reduce the viability of the MNTI cell line

Cells in the exponential growth phase were seeded in 96-well plates at a density of 3000 cells per well in a final volume of 100 μL and were treated, in quadruplet, with a single dose of 47 small molecule inhibitors targeted against proteins involved in epigenetic processes. Viability was measured at days 3, 7 and 10 using PrestoBlue (Life Technologies) according to the manufacturer’s protocol and normalized to vehicle controls. Day 3 viability was determined from triplicate readings and viability on days 7 and 10 from duplicate readings.

### Assay of sensitivity of cell lines to the specific Cdk4/Cdk6 inhibitor palbociclib

Cells were seeded in 96-well plates at a density of 2000 cells per well and were treated with doses of palbociclib (Cambridge Bioscience, Cambridge, UK) in the range of 0.1 to 30 μM. After 72 h, viable cells were assayed by adding CellTiter 96 AQueous One Solution Cell Proliferation MTS reagent (Promega, Southampton, UK) and measuring the absorbance of the formazan product at 490 nm. Log dose–response curves were obtained by fitting a four-parameter logistic function to the data (R 3.3.2, R Foundation for Statistical Computing). Mean IC_50_ values were derived from at least three independent experiments.

## Additional file

Additional file 1: Final list of candidate fusion genes with genomic coordinates and sequences output from FusionCatcher. (CSV 105 kb)

## Abbreviations

ALL: Acute Lymphoblastic Leukemia
BAM: Binary Short DNA Sequence Read Alignments File
BWA: Burrows-Wheeler Aligner Package
CNV: Copy Number Variations
FPKM: Fragments Per Kilobase Per Million Mapped Reads
GATK: Genome Analysis Toolkit
GSEA: Gene Set Enrichment Analysis
hNCC: Human Neural Crest Cells
miR: microRNA
MNTI: Melanotic Neuroectodermal Tumor of Infancy
SNP: Small Nucleotide Polymorphism
SNV: Single Nucleotide Variants
